# Children’s friendships in super-diverse localities: Encounters with social and ethnic difference

**DOI:** 10.1177/0907568216633741

**Published:** 2016-07-24

**Authors:** Humera Iqbal, Sarah Neal, Carol Vincent

**Affiliations:** University College London, UK; University of Surrey, UK; University College London, UK

**Keywords:** Ethnicity, friendship, identifications, social class, superdiversity

## Abstract

This article explores how children make, manage, or avoid friendships in super-diverse primary school settings. We draw on interviews and pictorial data from 78 children, aged 8–9 years across three local London primary schools to identify particular friendship groupings and the extent to which they followed existing patterns of social division. Children in the study did recognise social and cultural differences, but their friendship perceptions, affections, conflicts and practices meant that the way in which difference impacted relationships was partial and unstable. Friendship practices in the routine settings of school involved interactions across difference, but also entrenchments around similarity.

## Introduction

Friendships are an integral part of personal social and emotional worlds, a key site in which individuals learn to negotiate public social worlds – interacting, forming opinions, engaging in practices and co-existing with others. Friendships made in primary school are especially important given that they are some of the first occasions in which children begin to develop understandings of social and ethnic as well as individual difference. We argue that those primary schools situated in urban ‘super-diverse’ (Vertovec, 2007; Vertovec and Nowica, 2014) environments, in which local populations are defined by the complexity of varied ethnicities, economic backgrounds, migration histories and legal statuses, have the potential to be one of the few sites to facilitate friendships between adults and between children from different social class and ethnic backgrounds. These institutions bring parents and children together over a sustained period of time and with a shared focus of interest (the school). Here, we consider the processes of children’s friendship-making in three super-diverse primary school settings.

The data forms part of a larger study investigating adults’ and children’s friendships across social and ethnic difference. The extent to which living in diverse localities and routinely sharing social resources, such as schools, impacts intimate personal social relationships is explored by the project. Given that primary schools can act as a nucleus of contact making and friendship-making between adults and between children, the project explores in particular how the friendships children do and do not make impact adult (parent) relationships with other parents, and how children and adults conceptualise these friendships. In policy and school settings, relations such as friendship are often assumed to have an informal social cohesive property, enabling them to bring individuals together despite differences in their backgrounds, thereby facilitating ‘social mix’ and bridging different worlds (Hollingworth and Archer, 2010). This assumption has been investigated particularly with adolescents, probing their interactions and engagement at a societal level in both neighbourhoods and institutions (e.g. Harris, 2014; Hollingworth and Mansaray, 2012). A literature on ethnicity, younger children and social interactions also exists (see below). However, less is known about children’s interactions and perceptions of difference in their friendship relationships and practices in settings with highly complex, super-diverse populations. This article contributes to the existing literature through a focus on super-diverse settings; it begins by situating itself in literature around children forming social relations across social class, and ethnicity in school settings, before detailing the project’s design and methods. Following this, we present data on three themes: first, we briefly note the way in which social class and ethnicity impact the friendships across the three classes, but also point to the commonalities that bind the children together in the shared spaces of their classroom, playground and school. Second, we focus on ethnicity and social-class difference – the central concerns of our research – and note that relationships across ethnicity are largely unremarked, whereas the children seem to understand ‘difference’ much more in terms of materialities, such as possessions. Our third theme explores the mobile and emergent nature of children’s identities and identifications through a focus on religion. We conclude that children in settings with a mix of social, ethnic and cultural backgrounds recognise differences in often sophisticated and unexpected ways.

## Children’s friendship relations across ethnic and social difference

There is a strong association between children’s friendship formations and key adults in a child’s life – for example, teachers and parents – who can and do influence children’s friendship networks (see Corsaro, 2006; James et al., 1998; Vincent et al., 2016). However, researchers in the field argue for the centering of the autonomy of children in their decision-making round social relationships (Corsaro, 2006; George, 2007; Holloway et al., 2010; James, 1993, 2013; Smart et al., 2001). This is also the position taken by the authors of this article. Our data presented here highlights the agency of our respondent 8- to 9-year-olds and their own understandings of diversity and difference in relation to friendships, while also acknowledging the role of teachers and parents in shaping those friendships, for example, through their classroom management strategies (teachers) and the organisation of the children’s out of school time (parents/carers). In doing so, we also recognise the importance of context and how ethnicity and class are complex and their influences vary across different settings and circumstances (Huber and Spyrou, 2012; Morrow and Connolly, 2006).

Literature around friendship across social and ethnic difference in young people has tended to focus more on adolescents and young adults (e.g. Harris, 2014; Hollingworth and Mansaray, 2012; Rhamie et al., 2012) than on pre-teenage children. This research has explored friendship processes in schools, colleges, estates, neighbourhoods and communities in a range of cultural contexts. That schools and colleges are a re-occurring site of research attention reflects their capacity to ‘bring together’ and their proximate assembling of often diverse local populations. In this way, some schools serve as a setting for the exploration of the ‘compulsory’ encounter of difference. The extent to which young people navigate and manage levels of diversity in their day-to-day settings can involve cultural openness and hybridity as well as ‘harder lines’ of cultural defensiveness (Harris, 2014). Some studies of adolescents suggest that there is some, but limited, social mixing across ethnicity and particularly class (e.g. Hollingworth and Mansaray, 2011; Rhamie et al., 2012), but examples of work on primary schools with ethnically diverse pupils evidences young children engaging in cross-difference friendships. For example, Irene Bruegel’s (2006) study found that young children’s friendships can and do cross ethnic divides and she suggests that it is sharing daily routines which lead to such relationships, rather than more artificial initiatives such as twining schools. In her later work (Weller and Bruegel, 2009), she emphasises the role children play in the generation of neighbourhood social capital, directly through their own local relationships, and indirectly as parents come to form new networks around their children . Similarly, Knifsend and Juvonen (2013) used the construct of social identity complexity (Roccas and Brewer, 2002) to examine friendship groups among 11-year-olds in ethnically diverse schools in the United States. In the study, social identity complexity describes the overlap between social groups with which children identify, based on common interests such as football and fashion. They found social connectedness across ethnic groups increased when children perceived a connection of interests. Correspondingly, Sedano (2012) in an in-depth study of ‘Moroccan’ and ‘Gypsy’ 7- to 13-year-old children in Andalucía argues that ‘ethnicity is not a divisive force in children’s friendships, rather it is the gradual construction of shared cultural patterns’ (p. 382) that influences friendship making which allowed more recent migrant arrivals to socialise after their initial ‘strangeness’ in the eyes of their classmates had dissipated.

Young children’s cognitive recognition of difference is core to Connolly’s (1998) work on young children and patterns of division. In *Racism in Children’s Lives*, he evidences the extent to which children as young as 5 years, in an urban, ethnically mixed, working-class area very much recognised, ascribed, and engaged in racialised distinctions between themselves. Here, Connolly (1998) highlights the intersection of numerous contextual factors in the development of identities in children:

Their identities were therefore not simply determined by their age but also by their ethnicity, gender, class and sexuality. These all came together within specific contexts to provide the background against which the children developed their sense of identity. (p. 187)

In later work in the context of the conflicts in Northern Ireland, Connolly et al. (2009, 2011) continues to explore the ways in which children are aware of social divisions and may actively mobilise and make sense of their worlds through racialised and ethnicised notions of difference. For Connolly, children’s early years are not a period of being oblivious to difference, but the opposite – a critical time in which identifications around ethnicity form. Connolly argues that children were not simply repeating racism overheard and learnt from adults, but more actively reworking adult racialised discourses to make sense of their own social worlds and relationships. Given this degree of agency, and difference as a sense-making ‘resource’, Connolly notes various initiatives to work with and talk to young children about diversity in order to find ways to ‘unthink’ racialisations and negative perceptions of difference by young children. He describes a ‘multilayered’ (Connolly, 2006) approach, needed to conceptualise children’s lived experiences in which broader structural and institutional forms of racism but also more micro and interpersonal manifestations exist. Fox and Miller-Idriss (2008) build on this work stressing that it is ‘everyday ethnicity’ (p. 538) which needs to be studied, that is, the everyday life of a child and how they exhibit identities and belonging in different contexts and in their relationships with different people. In another study, Sime and Fox (2015) argue that children’s inter-ethnic networks post migration are influenced by age, class, gender, language, as well as perceptions of trustworthiness and cultural stereotypes.

These literatures highlight the extent to which social divisions and difference are recognised, known and used in children’s worlds and their friendship relations and practices. In our analysis below, based in settings comprised of highly complex mixes of social and ethnic diversity, we focus on the ways and extent to which this complexity is routinely negotiated by young children. We now turn to the design details of this analysis.

## The project’s methods and data sources

Data presented here draw on a larger study of children’s and adult’s friendships, which comprises semi-structured interviews with children, their parents and their teachers in three London state primary schools with mixed-class and multi-ethnic populations. Here, data from 43 individual and paired interviews with 78 Year 4 children (aged 8–9 years) from three primary classrooms, and their ‘social maps’ depicting their friendship networks are presented.

The three primary schools, *Leewood, Fernhill* and *Junction*, were carefully selected for the degree of ethnic and social mix. A commonality in each of the neighbourhoods was the existence of social housing and expensive private housing side by side, causing a mixed-class population to feed into the local primary schools. The localities were all experiencing different degrees of gentrification with Leewood being in the area of most established gentrification (see Neal et al., in press). Each of the three schools had been graded ‘good’ by Ofsted^1^ in 2013. The percentage of children on Free School Meals (a proxy indicator of relative poverty) differed across the schools, being lowest in Leewood (19.7%), reflecting the surrounding affluence of the neighbourhood. Percentages were similar for Junction (32.8%) and Fernhill (38.7%).^2^

The sample, across the schools, comprises children from a range of economic backgrounds and migration histories. Table 1 summarises details of the classroom groups, including their numbers and main ethnic compositions. The complex diversification of ethnicity within the sample, reflecting the super-diverse nature of the localities, is clear. We must note that we have relied on self-description of ethnicity wherever possible.^3^ However, the children described social class in such varied and inconsistent ways that we have chosen to present social-class data based on parental occupations, and only where we had that information.

**Table 1.** Details of the children’s ethnic backgrounds.

Ethnicity	LEEWOOD SCHOOL: Crimson class	JUNCTION SCHOOL: Burgundy class	FERNHILL SCHOOL: Scarlet class	Total
White British	13	4	2	19
White Other	1	7	3	11
	*1 Albanian*	*2 Cypriot*	*1 Romanian*	
		*3 Columbian*	*1 Portuguese*	
		*1 Bulgarian*	*1 French*	
		*1 Czech*		
Black (African/Caribbean/Other Black)^a^	8	9	9	26
	*3 Black Caribbean*	*5 Black Caribbean*,	*3 Black African*	
			*6 Black*	
	*5 Black African*	*4 Black African*	*Caribbean*	
South/East Asian	1	5	1	7
	*1 Indian*	*3 Bangladeshi*	*1 Indian*	
		*1 Pakistani*		
		*1 Chinese*		
Turkish/Kurdish	3	–	2	5
	*3 Turkish*		*1 Turkish*,	
			*1 Kurdish*	
Mixed	4	2	2	8
	*1 White/Black Caribbean*,	*1 Black African/White British*,	*2 Caribbean/White British*	
	*1 Indian/Anglo Indian*,			
		*1 Greek/Jamaican*		
	*1 White/Malay*,			
	*1 Eurasian*			
Arab	–	1	1	2
		*1 Algerian*	*1 Algerian*	
N	30	28	20	78

a Within this category (based on parents country of origin), there were (5 Somali, 1 Ethiopian and 1 child from Somaliland. The rest identified as Black African or Black Caribbean).

From the outset, the researchers clearly acknowledged the relationship between knowledge and power when both engaging with the children and analysing their narratives (Alanen and Mayall, 2001). In order to obtain these narratives, sustained research with children is important, yet, gaining access to children’s worlds and acquiring trust is challenging, given that adults are physically larger than children, possess more power and are often positioned as controlling of children’s behaviour (Corsaro, 2006).

Tied into this are arguments around positionality and the identities of the researchers. ‘Insider’ positioning in a super-diverse sample can be challenging given the plethora of cultural, linguistic, ethnic, national and religious identities. Each of the researchers were middle-class women, two White British and one British South Asian. There was a combined experience on research around multiculture, families and social class. Furthermore, extensive field-notes taken during the course of data collection ensured a reflexive stance was maintained by the researchers around their position and role in the research.

From early on, we attempted to position ourselves as separate from the teacher and other adults within the classroom (who represented adult authority). By first observing and informally chatting to children for several weeks, learning about their routines and developing trust before the interviews took place, we obtained rich data from the children, and were able to establish strong bonds with them over a short period. This was evidenced by the breadth and depth of subject matter they shared with us detailing their lives and their friendships. This also placed the children as active agents and knowledge producers. We had obtained parental consent through sending home letters describing the research and asking for permission to include their children. Parental consent forms were translated into a range of languages based on guidance from the class teacher. We were also careful to emphasise with the children that participation was voluntary. Before all the interviews we gave the children a consent form, written in accessible language, which emphasised the confidentiality of our discussions, and their right to stop at any time. We consulted with class teachers about children’s English language abilities before the interviews, and there was no instance where an interpreter was required with the children (although this resource was available). Interviews, lasting about 45 minutes, mostly with pairs of children took place away from the classroom. The pairs were carefully selected in consultation with the teacher, and the children were asked to draw maps of their friendships. Using these, we were able to construct larger maps of the social relations across the entire classroom in each school. Similar ‘sociogram’-based methods have been used in other friendship research to map out friendship networks, and reciprocities within the classroom setting (particularly in social psychology, see Banerjee et al., 2011). The visual data also served as a prompt for discussion (and provided the children with an activity to lessen the ‘formality’ of the interview) and has also been a data source for understanding the networks within each classroom.

Our small scale, but intensive, design was necessary, we argue, if we are to be able to develop an understanding of the nuances of friendships as the participants navigated the social terrain of their classes. The transcribed data were analysed through a combination of hand and software coding techniques. While our initial theoretical categories drew from existing literature on children, and class, multiculture and diversity, social capital, social mix and friendships, our code book was derived from team discussions focusing on an initial sample of transcripts (LeCompte et al., 1993). The codes were refined and challenged through further engagement with and scrutiny of data. It is the findings from this process that the paper now details. Data selected represent typical and in some cases exceptional examples of what the children told us.

### Friendships in super-diverse classrooms

We used the children’s friendship maps in order to gain insight into the social networks across the three classrooms. Nearly all the children in the research had close friendships with others in their class who were from a different ethnic group to themselves (we defined ‘close’ as meaning among their ‘top five’ friends). There were close cross-class friendships, but fewer in number than cross-ethnic friendships. However, a majority had close friends – in their top five – who had a different social background to themselves. When we looked at who the children said their closest friend was, there were still a significant number of friendships across ethnic difference (nearly three quarters). There were far fewer ‘best friend’ friendships across class difference (just over a quarter of the children; see Vincent et al., 2015).

In all three classes, there were identifiable friendship groups. However, shared children’s classroom culture and play practices of music, computer games and playground games was a source of commonality and bonding, and meant that memberships of the different friendship groups were not completely fixed. Rather, they were fluid. In each of the Year 4 classes, the children were encouraged by staff, especially their class teacher to develop a shared identity as a member of their particular classroom, so that they were frequently hailed as a collective – ‘Crimson class’, for example. In addition to this wider shared classroom identification, there was a ‘loose’ set of affective connections which existed between the children around music, computer and playground games. Games were often heavily gendered, for example, football, (Renold, 2004), although football was also productive of mixing across social class and ethnicity among the boys and an occasional girl who played. However, some games mentioned by the children, such as a range of chasing and catching games, were more inclusive. School ‘crazes’ – those particular intense moments of collective desire and engagement around an object or practice – worked as ‘super-mixers’ bridging difference. Loombands – brightly coloured rubber bands which were woven into bracelets – were one such example at the time of the fieldwork in Scarlet class in Fernhill School. The relative cheap cost of the bands made this an accessible, inclusive enthusiasm. The process of making the bands and gifting them to others produced high levels of interaction and exchange across difference. In this way, children’s practices, their ‘doing things’, are effective in creating ‘bringing together’ moments. Askins and Pain (2011), writing about young people and a community art project, also found the role of the materials themselves and the processes of ‘doing’, allowed the significance of difference in the social dynamics of the group to diminish (see also Knifsend and Juvonen, 2013 above).

Similarly, Sedano (2012) drawing on Bourdieu’s concept of field as a socially structured space argues from her study that ethnic divisions were not always the divisive force for the children which the adults around them understood them to be. In comparison to the institutional ‘bureaucratic’ field (e.g. school) and the domestic field, she found ethnicity to be of far less importance as a structuring principle in the ‘play field’ where children have the most agency. The loombands provide an example here of the children’s agentic choices to mix across difference for the pleasure of a shared activity. Elsewhere, we have written about the influence of adults – parents and teachers – on the children’s social networks (Vincent et al.,2016 ).

### Recognising difference

The children did recognise and were aware of the high levels of cultural diversity that were part of their daily worlds. In their conversations with us and in our observations of classroom and playground interactions, the children appeared to perceive and interpret social diversity, including ethnic, religious and linguistic diversity as a given and very much part of everyday social life. The schools all encouraged explicitly celebratory sensibilities around diversity; each held International Evenings, for families who were encouraged to bring food from parents’ country of origin, celebrated a range of festivals and positively referenced children’s bilingualism’ (Vincent et al., 2016). These sensibilities were often articulated by the children in interviews. For example, Callum (a Black Caribbean origin child in Crimson class at Leewood School) explained to us that

Oh yeah like the school don’t really have a lot of the same cultures, [we are] all mixed. It is not really – like you wouldn’t say that there is like one popular culture here, because literally we are all mixed … you get to learn more about other people and how they live and stuff.

In her study of Hackney in London, Wessendorf (2014) uses the term ‘commonplace diversity’ to argue for a similar phenomenon where adults routinely experience ethnic difference, and this is viewed positively, but also as unexceptional. Diversity understood as ‘ordinary’ was also illustrated by many of the children’s accounts, but this is not to say they did not notice difference. Indeed, we found, like Connolly (1998, 2011), that children drew on their understandings of difference to interpret and make sense of their worlds. This took place through a range of sites and signs which were read through difference – dress, types of food, languages spoken, what children were allowed to do at home, the things/possessions other children had, holidays, pets and so on. The conceptualisation of difference by the children in our study counters the narrative of ‘social invisibility’ (see Hunter et al., 2012) which suggests that young children do not perceive social difference nor assign it any social significance due to their ‘innocent’, unformed nature.

The children’s friendship maps and accounts also demonstrated the influence of social divisions in their friendships. Direct discussion about ethnicity, race and/or class was less common, and certainly, the verbalising of explicit racism was rare and treated as a serious matter by all three schools. However, a few significant narratives emerged in conversation with the children. For example, a Turkish boy in Crimson Class (Leewood school) described a Black Caribbean boy (Daine) as being like a ‘monkey’ who needed to ‘go to the jungle’. Daine was on the autistic spectrum, and in him this was invisible condition. His frustration had manifested itself in the past as anger. We can see here that his ethnicity as Black Caribbean intersects with his disability, leaving him vulnerable to prejudice.

In a complex vignette, Krystina, a White European girl from a White middle-class girl group in Junction school (comprising Krystina, Beth and Gwen) discussed how Tyler, (mixed working-class boy – White and Black Caribbean) had wanted to play with them, however, he had been told by a group of older, non-White children in the playground not to mix with Krystina and her friends because they were White, and he was not:

We asked someone if we could borrow the hoola-hoop and they (older children) were playing and they said, ‘No’ and then [Tyler] said a bad word and then about the White people. And we were playing with white people so it kind of upset me and Beth and Gwen … then we just left them. (Krystina)

Krystina struggles with this negative racialisation of her friend and her as ‘White’ and the accompanying exclusionary behaviour by the other children. Although this was only one out of a few explicitly mentioned examples, clearly, underlying tensions and dynamics around race can play out in the children’s friendships, influenced by external factors (such as the group of older children).

Alongside ethnicity, social class as a site of difference was perceived by some children, although with a specific focus and understanding which positioned materialities as a key and common mode through which difference was noted. For example, how children spoke about housing was particularly interesting in relation to their understanding of social-class difference. Gabra, (Black African, Leewood) has a mixed friendship group in Crimson class, made up of girls from different ethnicities. One girl in the group, Pippa (White British) is from a highly affluent middle-class background and Gabra describes how she enjoys visiting her home (one of the larger houses in the locality), yet she relates feeling uncomfortable when Pippa visits her own home, a smaller council house, without a ‘palace bathroom’. Here, economic capital comes to the fore, as causing anxiety and highlighting difference between the friends:

Gabra: I like going to Pippa’s house because it feels a bit weird when Pippa comes to my house.

INT: Why does it feel weird?

Gabra: Because I don’t know what to do. […] And Pippa’s mum and dad’s room and there’s a bathroom like inside and it’s like a palace bathroom and it looks cool because the floor is like stone.

INT: Marble?

Gabra: Yeah, marble and it looks so cool.

Gabra’s account of social difference speaks to recent culturalist turn in social-class debates (Savage et al., 2013; Tyler, 2013) which have emerged due to the intensifying social inequalities relating not only to wealth and income but social and cultural indicators such as educational attainment, housing conditions, and forms of leisure participation. Each of these serves as means of capturing the role of social and cultural processes in generating class divisions.

An understanding or ‘seeing’ of difference based on materiality was the mode in which social class and ethnic diversity was most recurrent in children’s accounts of difference. Morrow (2001) also points to this relationship between social capital and symbolic capital in being points of negotiation in children’s friendships. Symbolic markers such as particular material possessions (e.g. branded goods) can provide a sense of belonging or familiarity and thus facilitate social relationships. In an exchange between two White middle-class children in Leewood, the possession of the latest technology was a point in which the children were able to find commonality:

Ethan: So I have an iPod and a laptop […].

Megan: I have got an X box.

Ethan: The same!

Megan: And I have got a DS.

Ethan: I have a DS!

Inequalities in access to class-based resources and dispositions, however, result in variations in lifestyle, causing different sets of visibilities and invisibilities and different forms of knowing. For example, during another discussion with children from Scarlet class (Fernhill School) between Serena (White middle class) and Cain (Caribbean/White heritage, working class), disparities in class-based resources could be clearly seen during discussions around local shops. Serena clearly was not aware of the supermarket chain ‘Iceland’ that offers low–cost frozen foods:

Serena: I live in [near] a big car park.

Cain: Yeah and then you have got Iceland there.

Serena: Iceland?

Cain: Yeah you have got Iceland all the way down there.

Serena: What’s Iceland?

Cain: I can’t believe she doesn’t know what Iceland is [directed at interviewer]

Serena: Is it a shop?

Cain: Yeah.

Serena: Oh I thought it was a funfair kind of place.

This type of account, which was fairly frequent from the children, highlights the difference in social worlds between the two children but also shows how children manage to navigate these differences – for example, Cain’s disbelief is directed to us rather than Serena herself and her imagining of Iceland as positive – ‘a funfair’ – avoids any culturalist stigmatising of social class (Tyler, 2013). We now consider further how children negotiate and explore difference.

### Mobile identifications and complex friendship practices

Holloway et al. (2010) discuss the importance of identity positioning and the consideration of performances of identity in young people’s lives particularly in a mixed classroom setting. We found that social class and ethnic markers, at times, infiltrate the discussions children had around identity with children making bids for credible status and cultural capital. Materiality was again a key site for these delineations to be made (see above, for example, with Ethan and Megan). However, in Junction school with its large number of Muslim children, religious identity was also significant. Being Muslim in Burgundy class at Junction school, was more frequently cited and discussed in the children’s interviews, than in Crimson or Scarlett class at the other two schools, where there were fewer Muslim children. In Burgundy class, among these children, going to after-school Quranic classes was common, and Eid (which took place during the fieldwork) was an exciting event. In contrast to anti-Muslim discourses circulating in the wider political and social world, in Burgundy class, Muslim identity was of high status, to the extent that one girl, not from a practising Muslim family, claimed that identity for herself. Kim (Greek/Jamaican heritage, working class) is in discussion with Abner (Black African), and the interviewer. Kim describes her best friend Mehreen who is of Muslim background. The children debate what it means to be a Muslim and its intersection with other identities:

Kim: [Mehreen] is kind of like [me], because we are Muslims she is my Islamic sister.

INT: Okay so is your mum Muslim and your dad Muslim?

Kim: My dad is not Muslim but my mum is.

INT: Your mum is Muslim?.

Abner: Yes her dad is from Jamaica, Jamaican African.

INT: So what do you mean by Islamic sisters?

Kim: That means Muslim sister.

INT: Yeah do you like being Muslim?

Kim: Yeah, some people like tease me and say ‘You are not a Muslim, you are not a Muslim’. I know I am a Muslim.

INT: Why would they say that to you?

Abner: That is what Sultan [Turkish boy in class] said to her.

Kim: Because I don’t celebrate things that I am supposed to. But some people didn’t.

Abner: You are not like a real Muslim like –

Kim: I am.

Abner: Not like – she is a Muslim but not like a strict one. The ones that are proper

Muslims, you are not like a proper Muslim. Like because your mum doesn’t wear a scarf everyday.

Kim appears to want to identify with being Muslim in order to bring her closer to her friend Mehreen. This exchange, which was one among a few, shows the children negotiating difference in order to produce high status identities. While Abner does not directly challenge Kim’s claim to be Muslim, he does not let her claim go entirely uncontested either – he will allow that she is a Muslim, just not a ‘proper’ one, as ‘proper’ Muslims have visibly identifiable markers (e.g. headscarf). The exchange highlights the dynamic nature both of identities – in this instance religious identity – and the processes of identification that children engage in as they emphasise particular identity markers in different contexts, allowing them to develop affective relationships with a range of peers. This idea of shifting identities in a mixed classroom around Islam is also highlighted in the work of Zine (2001). Furthermore, Huber and Spyrou (2012) describe this ability of children to cross borders, form new boundaries and blur boundaries as being a means of appropriating, reinterpreting or subverting dominant norms. They argue that the trading of different discourses by children could allow for them to cope with stigma and ethnic stereotype and produce more inclusive identities.

Given the diverse nature of Muslim groups around the world, it is perhaps not surprising that the children also had different ideas about a ‘proper’ Muslim identity. Here, Arzu (Turkish Cypriot) describes how mixing across ethnic difference can sometimes be challenging and she stereotypes the other Muslim children as ‘all enjoying rice’, while she herself does not. In doing so, she distances herself from the practices of ‘strict Muslims’, and perhaps (though not explicitly) makes a bid to a high socio-economic status given the associations between rice as a dietary staple and poverty:

INT: Do people in the class have birthday parties ever?

Arzu: Yes, but not Jamilah or Amina because they are Muslims so they don’t have like a party.

INT: Are you not allowed to have a birthday party if you are a Muslim?

Arzu: But I could but they are strict Muslim and my mum says I’m not a strict Muslim, that’s why.

INT Okay so if you are a strict Muslim are you not allowed to have a birthday party?

Arzu: No. Only you are allowed to go to Eid.

INT: Are there lots of Muslims in the class?

Arzu: Yes there are loads, that is why they do rice.

INT: They do what?

Arzu: They do rice for lunch because there are loads of Muslims that’s why.

INT: Okay so Muslims eat rice?

Arzu: I don’t want rice, I don’t feel like it, that is why.

The exchange reflects an intriguing cultural geometry on the part of Arzu as she distances herself from ‘strict Muslim’ practices and mobilises materiality (rice) and practices (birthday parties) as the sites through which her own Muslim identity is expressed. Like Connolly (1998), we would also suggest that the social competencies of young children allow them to reflect on and intervene in their own social worlds. As noted above, from his study, young children in inner city multi-ethnic schooling Connolly argues that some young children were able to ‘appropriate, rework and reproduce discourses on ‘race’, gender and sexuality in quite complex ways’ (p.187). This highlights the agency and ability of children to interact within different social environments and adapt their behaviour according to different contexts.

## Concluding reflections

Investigating the friendships of children in super-diverse primary school settings allows us to explore how children from varied social, religious, ethnic and racial backgrounds negotiate the complexities of their particular micro-environments. The children viewed diversity as an ordinary part of their everyday lives, but at the same time, as something that is the subject of celebratory phenomenon by their schools. These two positionings – the ordinary and the celebratory – make up a social terrain which they are required to negotiate and manage. We have suggested that the children drew on a multidimensional awareness of difference to make sense of their worlds and friendship relations. Our work chimes with Connolly’s argument that young children are very much engaged in identification processes which include conceptions of ethnicity and class. However, unlike the more ‘extreme’ disadvantaged and/or conflict environments in which Connolly’s work is located, the super-diverse localities in the project meant that most of the children in our project were surrounded by intense levels of social, ethnic and religious differences and were themselves constituents of these complex populations with their own complex identities. In this context, the children in the study appeared to be able to participate across difference in proto-skilled ways, to generally mix competently and without major tensions or frequent recourse to racialisation and othering. In this way, our findings speak to those of Weller and Bruegel (2009) in relation to primary school children’s capacities to manage and interact with social and ethnic difference in their school worlds. We would stress that these capacities were partial and uneven. A co-existence of mixings and separations (Harris, 2014) prevailed, such that instances of social and ethnic interactions, of friendship formations across difference were also coupled with relationships in which children’s friendships appeared to be ones of affinity towards those socially and ethnically similar to themselves.

We have argued that materialities and practices inform children’s recognitions of differences but points of commonality are also part of the ways in which differences are negotiated and diminished. This highlights how context and situation are very much part of friendship relations. The space in which friendships were played out was also of relevance in the children’s worlds (also Sedano, 2012 above). Friendships inside the classrooms were not always replicated outside the classroom, and parental management of children’s out of school friendships also influenced mixing (see author reference). It was also clear that mixing across class distinctions occurred less frequently than mixing across ethnicity, and that gender and religion were other important factors in the children’s friendship configurations.

What emerged was the ability of children to engage in a process of identity building and management; learning to ‘define’ themselves in different ways in different contexts (Allan, 2011). However, this process is partial – the children do not have complete freedom here, as their peers, parents and teachers are all engaged in additional readings of them, their social relationships and perceived social competencies. It is in this context that we note that children’s friendships are not independent of the adult world surrounding them. However, despite this, the ability of the children to negotiate different identities according to setting and context allowed for the management of friendships across difference, and we have focused on religious identities as an example here.

We can conclude that children in the study could and did form friendships across class, ethnic and religious difference, but that their practices of friendship-making also reflect the influence of dimensions of social division.

## Notes

1. The English regulator of schools.

2. The national average in state-funded nursery and primary schools in 2013 was 19.2%.

3. Adult interviewees all completed a self-description form of ethnicity and social class for themselves and their children. The children voluntarily self-described their ethnicity and all identified their parents’ country of origin despite the majority of the children being born in London.
